# The Podovirus ϕ80-18 Targets the Pathogenic American Biotype 1B Strains of *Yersinia enterocolitica*

**DOI:** 10.3389/fmicb.2020.01356

**Published:** 2020-06-19

**Authors:** Karolina Filik, Bożena Szermer-Olearnik, Maciej Wernecki, Lotta J. Happonen, Maria I. Pajunen, Ayesha Nawaz, Muhammad Suleman Qasim, Jin Woo Jun, Laura Mattinen, Mikael Skurnik, Ewa Brzozowska

**Affiliations:** ^1^Hirszfeld Institute of Immunology and Experimental Therapy, Polish Academy of Sciences, Wrocław, Poland; ^2^Department of Microbiology, Institute of Genetics and Microbiology, Faculty of Biological Sciences, University of Wrocław, Wrocław, Poland; ^3^Department of Biosciences, Institute of Biotechnology, University of Helsinki, Helsinki, Finland; ^4^Research Programme Unit Immunobiology, Department of Bacteriology and Immunology, Human Microbiome Research Program, Faculty of Medicine, University of Helsinki, Helsinki, Finland; ^5^Molecular and Integrative Biosciences Research Programme, Faculty of Biological and Environmental Sciences, University of Helsinki, Helsinki, Finland; ^6^Department of Aquaculture, The Korea National College of Agriculture and Fisheries, Jeonju, South Korea; ^7^Division of Clinical Microbiology, Helsinki University Hospital, HUSLAB, Helsinki, Finland

**Keywords:** bacteriophage, *Yersinia enterocolitica*, phage biocontrol, phylogenetic, podovirus, proteome, genome

## Abstract

We report here the complete genome sequence and characterization of *Yersinia* bacteriophage vB_YenP_ϕ80-18. ϕ80-18 was isolated in 1991 using a *Y. enterocolitica* serotype O:8 strain 8081 as a host from a sewage sample in Turku, Finland, and based on its morphological and genomic features is classified as a podovirus. The genome is 42 kb in size and has 325 bp direct terminal repeats characteristic for podoviruses. The genome contains 57 predicted genes, all encoded in the forward strand, of which 29 showed no similarity to any known genes. Phage particle proteome analysis identified altogether 24 phage particle-associated proteins (PPAPs) including those identified as structural proteins such as major capsid, scaffolding and tail component proteins. In addition, also the DNA helicase, DNA ligase, DNA polymerase, 5′-exonuclease, and the lytic glycosylase proteins were identified as PPAPs, suggesting that they might be injected together with the phage genome into the host cell to facilitate the take-over of the host metabolism. The phage-encoded RNA-polymerase and DNA-primase were not among the PPAPs. Promoter search predicted the presence of four phage and eleven host RNA polymerase –specific promoters in the genome, suggesting that early transcription of the phage is host RNA-polymerase dependent and that the phage RNA polymerase takes over later. The phage tolerates pH values between 2 and 12, and is stable at 50°C but is inactivated at 60°C. It grows slowly with a 50 min latent period and has apparently a low burst size. Electron microscopy revealed that the phage has a head diameter of about 60 nm, and a short tail of 20 nm. Whole-genome phylogenetic analysis confirmed that ϕ80-18 belongs to the *Autographivirinae* subfamily of the *Podoviridae* family, that it is 93.2% identical to *Yersinia* phage fHe-Yen3-01. Host range analysis showed that ϕ80-18 can infect in addition to *Y. enterocolitica* serotype O:8 strains also strains of serotypes O:4, O:4,32, O:20 and O:21, the latter ones representing similar to *Y. enterocolitica* serotype O:8, the American pathogenic biotype 1B strains. In conclusion, the phage ϕ80-18 is a promising candidate for the biocontrol of the American biotype 1B *Y. enterocolitica.*

## Introduction

*Yersinia enterocolitica* is a gram-negative bacterium that belongs to the *Enterobacteriaceae* family. It is a human enteropathogen (Thomson et al., 2006). *Y. enterocolitica* strains are classified into six biogroups based on phenotypic characteristics, and to 57-O serogroups based mainly on difference in the lipopolysaccharide (LPS) O-antigen structures (Fàbrega and Vila, 2012). Yersiniosis is a zoonotic foodborne infection of animals and humans caused by pathogenic strains of *Y. enterocolitica* that mainly belong to bioserotypes 1B/O:8, 2/O:5,27, 2/O:9, 3/O:3, and 4/O:3. The strains of bioserotype 4/O:3 cause the majority of the infections in Europe, Japan, Canada and the United States (Bottone, 1999; Fredriksson-Ahomaa et al., 2006). In Europe and China, the most prevalent are the *Y. enterocolitica* serogroups O:3 and O:9, whereas in United States the predominant serogroup is O:8 (Sabina et al., 2011). In recent years, *Y. enterocolitica* infections have also spread between continents through human travel and transportation of pigs, and this has resulted in the higher occurrence of *Y. enterocolitica* O:8 infections in Europe (Rastawicki et al., 2009) and also in Japan (Ichinohe et al., 1991). The main reservoir of pathogenic *Y. enterocolitica* are pigs, and infections are caused especially by consumption of raw or undercooked pork, but dogs have also been implicated as a potentially significant source in rural communities. In addition, direct and indirect contact with feces from contaminated livestock can also lead to infection (Sabina et al., 2011; Wang et al., 2011). In humans, the infection is usually localized to the gastrointestinal track and the bacteria may also cause mesenteric lymphadenitis. The most common symptoms of the infection are acute enteritis, fever, vomiting, inflammatory and watery diarrhea (Fàbrega and Vila, 2012).

The pathogenic *Y. enterocolitica* strains are characterized by the presence of virulence factors encoded by the genes located either in the chromosome or in the 70 kb virulence plasmid, pYV. The most important virulence factors are the LPS, the adhesins/invasins (Inv, YadA, Ail), the flagella, the type 3 secretion system (T3SS) and the enterotoxin Yst. These virulence properties help *Y. enterocolitica* bacteria to survive and colonize the human host and cause the symptomatic infections (Fàbrega and Vila, 2012).

The invention of antibiotics has certainly saved millions of lives, but currently the rapid acquisition of antibiotic resistance by bacteria has become a major epidemiological problem. According to World Health Organization, antibiotic resistance is one of the biggest threats to global health and food security. Therefore, we have to take into use alternative approaches to combat the drug-resistant bacteria.

Bacteriophages are the most abundant organisms on Earth. The total number of phages has been estimated to be around 10^31^ particles (Hendrix, 2002; Leon-Velarde et al., 2019). The therapeutic potential of phages was recognized in the early twentieth century; specifically in the 1930s and 1940s (Fischetti et al., 2006). Lytic phages have been used as therapeutic and prophylactic agent in controlling bacterial infections (Jun et al., 2018). Phage therapy is becoming an interesting being an alternative to antibiotic therapy. Since Alexander Fleming’s discovery of antibiotics, the overuse of antibiotics has imposed selective pressures on microorganisms. This has caused microorganisms to develop resistance mechanisms such as enzymatic mechanisms of drug modification, enhanced efflux pump expression, mutated drug target, etc. (Alekshun and Levy, 2007). During the last 10 years, phage research has become very popular especially scientist are now focused on the genome and evolution of bacterial viruses as well as horizontal gene transfer (HTG) which is the main cause of diversity (Leon-Velarde et al., 2019).

Bacteriophages characterized by exceptional specificity and selectivity, can only infect and reproduce inside the host bacteria (Ventola, 2015; Zhao et al., 2019). This specificity makes them an excellent tool to fight the pathogenic bacteria and it also provides a number of possibilities for diagnostic applications. Therefore, learning about the biology of bacterial viruses is an important research topic. Bacteriophages have long been utilized as tools in bacterial genetics and systematics. Indeed, the first suspicions that the genus *Yersinia* belongs to the Enterobacteriaceae was made on the basis of common sensitivities to phages (Brubaker, 1972). Phages have also been used in epidemiological characterization and other studies on *Y. enterocolitica* strains (Nicolle et al., 1967; Baker and Farmer, 1982).

Several bacteriophages infecting *Y. enterocolitica* have been isolated and characterized in the Skurnik laboratory (Skurnik, 1999). By using different host strains for enrichment, phages with different specificities were obtained and several of them were shown to use different parts of the *Y. enterocolitica* LPS as receptor (Skurnik, 1999). Detailed characterizations of several bacteriophages have been published including the T3-related ϕYeO3-12 (Pajunen et al., 2000, 2001; Kiljunen et al., 2005b), the giant myovirus ϕR1-37 (Kiljunen et al., 2005a; Skurnik et al., 2012; Leskinen et al., 2016), and the T4-like myovirus ϕR1-RT (Leon-Velarde et al., 2016). Genetic and structural data showed that the surface receptors of phages ϕR1-37 and ϕYeO3-12 are the outer core (OC) hexasaccharide and the O-antigen of the *Y. enterocolitica* O:3 LPS, respectively (Al-Hendy et al., 1991; Skurnik, 1999; Pajunen et al., 2000; Pinta et al., 2010; Skurnik et al., 2012), and that phage ϕR1-RT uses both OmpF and LPS inner core as receptors (Leon-Velarde et al., 2016).

In this paper, we describe the characterization of the *Y. enterocolitica* serotype O:8 specific phage ϕ80-18 that was isolated in 1991 and used as a tool in genetic selections (Zhang and Skurnik, 1994). We have earlier shown that purified O:8 LPS inhibits the phage and that the phage can infect an *E. coli* strain expressing the *Y. enterocolitica* serotype O:8 O-antigen, confirming that the O:8 O-antigen is the host receptor of ϕ80-18 (Zhang and Skurnik, 1994; Zhang et al., 1997). However, a detailed characterization of the phage has been missing and is presented here.

## Materials and Methods

### Bacteriophage, Bacteria and Culture Media

The bacterial strains used in this work are described in Supplementary Table S1. Isolation of the phage ϕ80-18 has been described earlier (Zhang and Skurnik, 1994). Both the phage ϕ80-18 and its host strain *Y. enterocolitica* serotype O:8 strain 8081-c have been deposited to Deutsche Sammlung von Mikroorganismen und Zellkulturen GmbH – Leibniz – Institut DSMZ under catalog numbers DSMZ 23253 and DSMZ 23249, respectively. Bacteria and bacteriophages were grown in lysogeny broth (LB, Bertani, 2004) at room temperature (22–25°C RT) unless otherwise indicated.

### Bacteriophage Propagation

*Yersinia enterocolitica* strain 8081-c was grown in LB for 16 h, and 0.1 ml of the culture added to 5 ml of LB. The bacteria were grown aerated at 28°C to exponential phase (OD_600_ = 0.3–0.5), 0.1 ml of a crude phage lysate (4.5 × 10^7^ PFU/ml) was added, and the culture was then incubated overnight at 28°C with shaking. The obtained phage lysate was filter-sterilized using a 0.22 μm Millipore membrane. In addition, a bacteriophage propagation experiment at 4, 28, and 37°C was performed, according to this scheme.

### Determination of Host Ranges and Efficiency of Plating

To evaluate the host range of the phage, the infectivity of the membrane-filtered phage lysate (10^8^ PFU/ml) was tested on the bacterial strains listed in Supplementary Table S1, using either the drop-test or plaque formation assay on soft-agar embedded bacteria. The formation of lysis zone or individual plaques was determined after 24 h of incubation. For the efficiency of plating (EOP), the PFU measurements were determined using the double-layer agar method. The EOP was calculated as the ratio between the PFU of the test strain to that of the original host strain *Y. enterocolitica* serotype O:8 strain 8081 (Stor ID 1258, Supplementary Table S1). The EOP assays were performed in triplicate.

### Genome Sequencing, Assembly and Annotation

Phage DNA was obtained from high-titer phage preparations as described earlier (Sambrook et al., 2001). Phage DNA was sequenced using the Illumina GAIIx (Genome Analyzer) technology at the FIMM Sequencing unit (Helsinki, Finland). The sequence assembly was done with the NextGene^1^ and Staden software packages (Staden et al., 2003). The Artemis genome-browsing and annotation tool (Rutherford et al., 2000) was used for genome annotation. The physical ends of the phage genome and the terminal repeats (approx. 200 bp) of ϕ80-18 could not be identified from the *de novo-*assembled genomic sequence. To carry out this we used the approach described in details previously (Salem and Skurnik, 2018). Briefly, a 500 bp PCR-amplified fragment of the *fliC* gene of *Y. enterocolitica* O:3 was ligated with phosphorylated phage genomic DNA. The ligation mix was then used as a template for PCR using a primer pair of which one primer was *fliC–*specific and the other primer one of the phage-specific primers predicted to be close to the physical ends of the phage genomes as described (Salem and Skurnik, 2018). The resulting PCR products were purified and sequenced using a *fliC –*specific nested primed located ca. 200 bp upstream of the ligation junction. The PSI-BLAST (Altschul et al., 1997) and HHPred (Söding et al., 2005) programs were used to identify homologous proteins. Genome identity analysis between different viruses was carried out using StretcherN at EBI (Li et al., 2015). The PHIRE search tool was used to identify phage-encoded RNA polymerase promoters (Lavigne et al., 2004). The sigma-70 specific bacterial promoters and rho-independent terminators were searched using the search tools BPROM and FindTerm, respectively (Solovyev and Salamov, 2011). The annotated genome sequence of phage ϕ80-18 has been deposited into the nucleotide sequence databases under the accession numbers HE956710 and NC_019911.2.

### Proteomics

Phage particle proteomes were analyzed by liquid chromatography coupled with mass spectrometry (LC-MS/MS) at the Proteomics Unit, Institute of Biotechnology, University of Helsinki. The phage with a titer >10^10^ pfu/mL was used for the analysis. Prior to digestion of proteins to peptides with trypsin, the proteins in the samples were reduced with tris (2-carboxyethyl) phosphine (TCEP) and alkylated with iodoacetamide. Tryptic peptide digests were purified by C18 reversed-phase chromatography columns (Varjosalo et al., 2013) and the mass spectrometry (MS) analysis was performed on an Orbitrap Elite Electron-Transfer Dissociation (ETD) mass spectrometer (Thermo Scientific, Waltham, MA, United States), using Xcalibur version 2.2, coupled to a Thermo Scientific nLC1000 nanoflow High Pressure Liquid Chromatography (HPLC) system. Peak extraction and subsequent protein identification were achieved using Proteome Discoverer 1.4 software (Thermo Scientific). Calibrated peak files were searched against all amino acid sequences of all six open reading frames of ϕ80-18 by a SEQUEST search engine. Error tolerances on the precursor and fragment ions were ±15 ppm and ±0.8 Da, respectively. Hits with at least two identified tryptic peptides were regarded as true hits.

### Electron Microscopy

The purified bacteriophage was applied to the surface of formvar carbon-coated copper grids and negatively stained with 2% uranyl acetate for 1 min. The excess of uranyl acetate was then removed from the grids using filter paper and the grids were allowed to air dry for 20 min (Ackermann, 2009). Preparations were visualized using a JEOL JEM-1200 EX 80 kV TEM. The dimensions of the bacteriophages were determined using RADIUS EM Imaging Software.

### Thermal and pH Stability Tests

To determine the thermal stability of phage ϕ80-18, phage samples (4.5 × 10^7^ PFU/ml) were incubated at 4, 25, 40, 50, 60, and 80°C for 2 h. Phage survival was determined from samples collected after 20, 40, 60, 80, 100, and 120 min incubation using the double-layer agar method (Chen et al., 2016; Zhao et al., 2019).

To determine the pH stability of phage ϕ80-18, 200 μl samples of the phage (4.5 × 10^7^ PFU/ml) were incubated under various pH conditions (2, 3, 5, 6, 7, 8, 10, and 12) for 2 h at 28°C. Bacteriophage preparations were mixed with different pH solutions in the volume ratio 1:1. Phage titers in the tubes were determined using the double-layer agar plate method.

### One-Step Growth Curve

*Yersinia enterocolitica* 8081-c bacteria, grown in 5 ml of LB to an OD_600_ of 0.5, were centrifuged at 12000 × *g* for 15 min at 4°C, and resuspended in 5 ml of fresh LB medium. The bacteria were then infected with phage ϕ80-18 at a MOI of 0.01, and the phages were allowed to absorb to the bacteria for 5 min at 28°C. To remove the unadsorbed phages the suspension was centrifuged at 14000 × *g* for 1 min, the bacterial pellet washed twice with fresh LB, and finally resuspended to 5 ml of LB, followed by incubation at 28°C. 100 μl from the sample were withdrawn from the tube every 10 min and the phage titers assayed using the double-layer agar method. The experiment was repeated three times (Zhao et al., 2019).

### Phylogenetics Analysis

The phylogeny of phage ϕ80-18 was determined using both the whole genome nucleotide and the RNA polymerase (RNAP) amino acid sequences for the analysis. The genomic sequences of representative *Autographivirinae* (taxid:542835) phages most closely related to ϕ80-18 (NC_019911.2) were identified using the BLASTN search. The genome-based phylogenetic tree was constructed using the VICTOR web service (Meier-Kolthoff and Göker, 2017) based on the Genome-BLAST Distance Phylogeny (GBDP) method (Meier-Kolthoff et al., 2013) and FastME software. This included 100 pseudo-bootstrap replicates and SPR post-processing (Lefort et al., 2015). The amino acid sequences of the most closely related RNAP proteins were identified using the BLASTP search. The sequences were aligned with MAFFT v7.429 under the L-INS-i strategy (Katoh and Standley, 2013). The best-fit model for tree reconstruction (LG+F+I+G4, chosen according to BIC) was calculated with ModelFinder (Kalyaanamoorthy et al., 2017). The RNAP phylogenetic tree was inferred by maximum likelihood method with IQ-TREE v1.6.11, performing ultrafast bootstrap with 1000 replicates for calculating branch support (Hoang et al., 2018). The phylogenetic trees were visualized with FigTree (Rambaut, 2006) and tanglegram was constructed with Dendroscope (Huson et al., 2007).

## Results

### Genome Analysis of ϕ80-18

**FIGURE 1:**
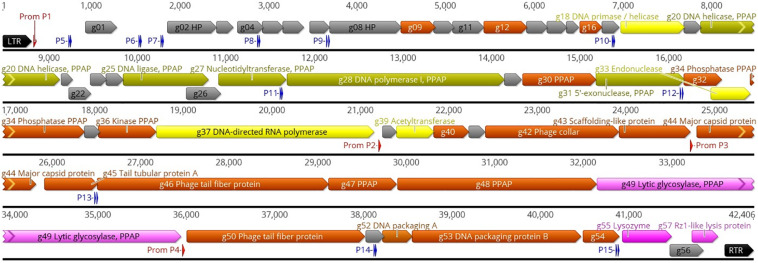
Genomic map of phage ϕ80-18. The nucleotide sequence of the phage is represented by the black horizontal line, above which are indicated the left and right terminal repeats (LTR and RTR, respectively) as black arrows, the phage RNAP promoters as red arrows, and the sigma-70 host RNAP promoters as blue double arrows representing the -35 and -10 boxes. The promoters are numbered and detailed information of them is given in Supplementary Table S3. All the predicted genes are indicated by different-colored arrows and the gene names and predicted functions are indicated either inside or outside the arrows. The genes encoding hypothetical proteins (HP) are gray. The genes encoding phage particle-associated proteins (PPAP) are dirty green (for genes predicted to encode enzymes) and brown (for genes encoding predicted structural proteins). The genes predicted to encode phage particle-associated lytic glycosylase, and the lysozyme, are pink, and the genes predicted to encode DNA primase, endonuclease and RNA polymerase are yellow. The map was produced using the Geneious 10.2.6 (www.geneious.com).

Phage ϕ80-18 has a linear double-stranded DNA genome of 42,406 bp with the GC content of 47,64% that is close to that of *Y. enterocolitica* strain 8081 (47%) (Thomson et al., 2006). Altogether 57 genes were predicted from the sequence, all in the forward strand (Figure 1). No tRNA coding genes were found. The physical ends of the genome contain 325 bp direct repeats (Figure 1). While the function of altogether 29 predicted gene products showed no similarity to any known genes in the databases and remained therefore unassigned, similarity searches by BLASTP (Altschul et al., 1997) and HHPred (Söding et al., 2005) assigned a putative function to 17 gene products. The remaining 11 predicted gene products were identified as phage particle-associated proteins (PPAPs) in the phage particle proteome analysis (Figure 1 and Supplementary Table S2). Altogether 25 PPAPs were detected by LC-MS/MS analysis including those identified as structural proteins such as major capsid (Gp44), phage collar protein (Gp42), scaffolding protein (Gp43) and identified tail component proteins (Gp45, Gp46, and Gp50) as well as the DNA packaging proteins A and B (Gp52 and Gp53). Also the peptidoglycan penetrating lytic murein transglycosylase protein (Gp49) was identified as a PPAP. The catalytic domain of the 1259 residue Gp49 occupies 150 N-terminal residues, thus it is likely that the remaining protein functions as a tape measure protein to determine the length of the tail tube that is extended upon adsorption of the phage particle on host bacteria (Hu et al., 2013). In addition, also the DNA helicase (Gp20), DNA ligase (Gp25), DNA polymerase (Gp28), and 5′-exonuclease (Gp31) proteins were identified as PPAPs, suggesting that they might be injected together with the phage genome into the host cell to facilitate the take-over of the host metabolism. In contrast, the phage-encoded RNA-polymerase and DNA-primase were not PPAPs. For the 56 predicted genes, the initiation codon was ATG and only for the *g37* gene encoding the DNA-directed RNA polymerase, it was GTG.

### Promoters

Using the PHIRE search tool, four 25 nt long phage promoters, designated P1 – P4 with a consensus sequence of -TGAT(T/a)(c/g)TCTACCCATATAG(c/t)AA(C/t)(A/t), typical for the *Autographiviridae*, were identified upstream the *g01, g38, g44*, and *g50* genes (Figure 1 and Supplementary Table S3). These promoters likely regulate the expression of the phage genes during different phases of the infection cycle. In addition, using the BPROM search tool for bacterial sigma-70 type promoters we identified 11 bacterial promoter candidates, designated P5 – P15 (Figure 1 and Supplementary Table S3). While the functionality of these promoters awaits experimental evidence, the highest scores were predicted to P5 located leftmost in the genome and very likely the first one to start transcription upon the injection of the phage genome into the bacterial cell. We did not detect the phage encoded RNA polymerase in the phage particle so it has to be synthetized *de novo* before the phage promoters can be utilized, therefore, the presence of eleven sigma-70 type promoters scattered around the phage genome will allow transcription of the necessary phage genes, including *g37* encoding the phage RNA polymerase. Only one rho-independent terminator was detected by the FindTerm program, located inside the *g37* gene encoding the phage RNA polymerase.

While in general, the genomes of many podoviruses can be divided into three regions comprising early, middle and late genes for virus-host interactions, DNA metabolism and virion structure and assembly, respectively (Wang et al., 2016), this classification could not be directly applied to phage ϕ80-18 genome. While the phage RNAP promoters P2, P3, and P4 all seem to direct the transcription of the late genes, only phage RNAP promoter P1 remains for the first half of the genome. Therefore, the sigma-70 type promoters that are scattered around the genome might be involved in the transcription of the early and middle genes. To the latter ones based on functional predictions would belong the genes *g18–g37* (Figure 1). Then the predicted early genes are *g01–g17*, among which are located also genes *g09, g12*, and *g16*, that encode PPAPs of unknown function.

### Phylogenetic Analysis of ϕ80-18

**FIGURE 2:**
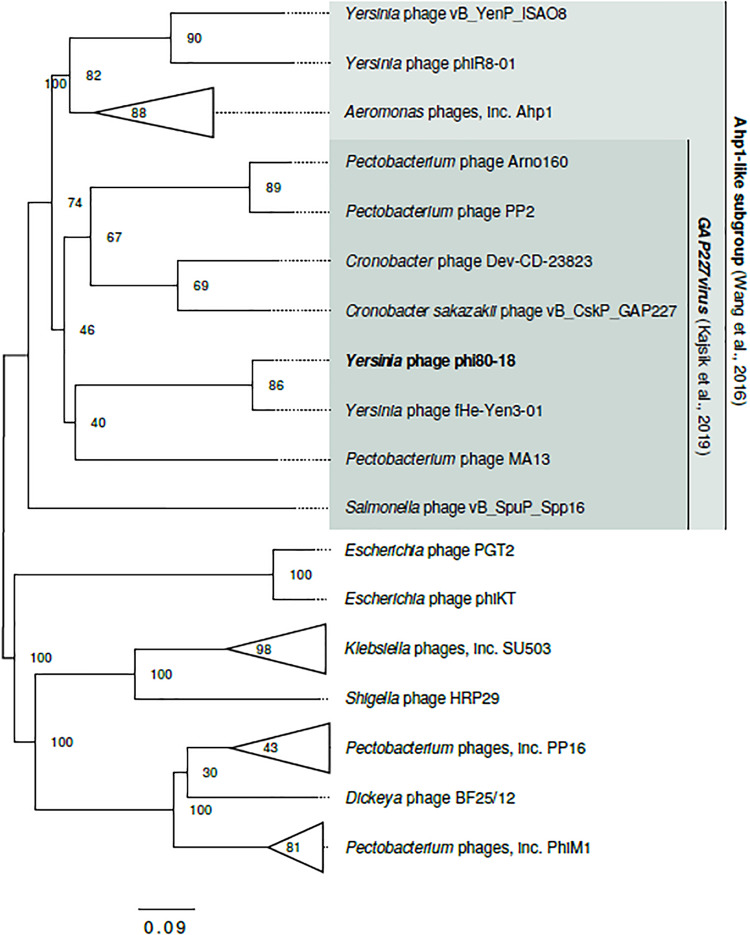
Genome-based phylogram of *Autographivirinae* representatives closely related to ϕ80-18. Node labels are bootstrap values. Ahp1-like subgroup (Wang et al., 2016) and *Gap227virus* genus (Kajsík et al., 2019) are proposed, yet not recognized by ICTV taxonomy (2018b Release). Here, both groups are extended beyond the range of taxa proposed in the original publications to cover the full cluster. The complete topology of the tree is shown in Figure 3.

**FIGURE 3:**
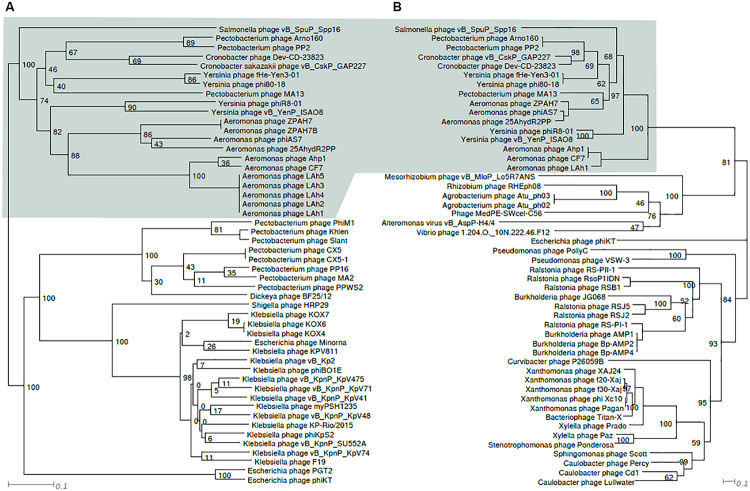
Tanglegram comparing topologies of genome based **(A)** and RNAP protein sequence based **(B)** phylograms of taxa closely related to ϕ80-18. Node labels are bootstrap values. The shaded area indicates similarly, composed clusters.

**FIGURE 4:**
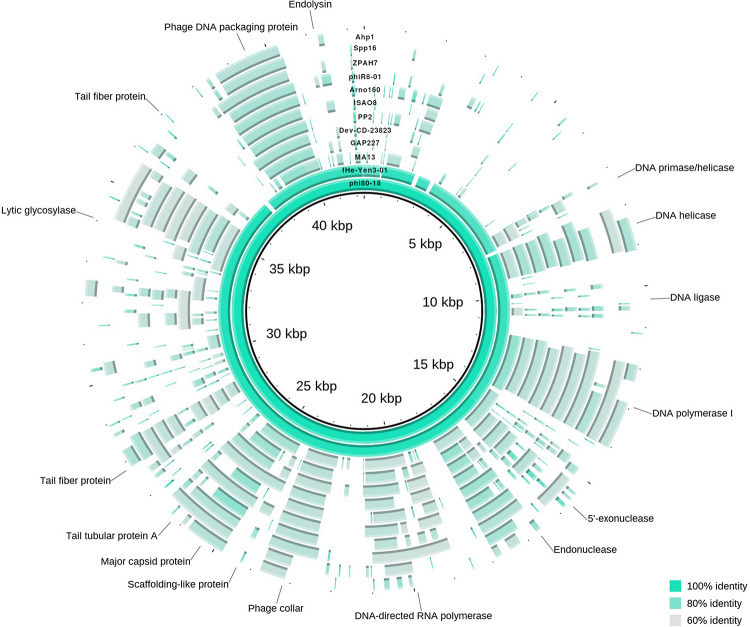
Genome map of ϕ80-18 and alignment to closely related bacteriophages according to phylogenetic analysis. The circular map represents the genome alignment of phages closely related to ϕ80-18, forming an unified cluster in phylogenetic tree (marked in Figure 2). Several predicted genes indicated with putative product names, the others remain as hypothetical proteins. Fragments, showing more than 60% identity with the ϕ80-18 genome sequence, are colored. The closest related phage, fHe-Yen03-1, scores over 92% of sequence identity and differ from ϕ80-18 only in minor traits (described in text). The other phages all more distantly related with below 75.5% identity. The most conserved genomic regions between the phages contain genes encoding DNA-processing proteins such as helicase, polymerases or DNA packaging protein.

The phage ϕ80-18 has been assigned to the *Podoviridae* family and the *Autographivirinae* subfamily like the model bacteriophage T7 or T3. BLASTN analysis revealed the highest sequence identity of 98 with 97% coverage (total identity of 93.2%, as determined by the EMBOSS stretcher alignment tool) to another *Yersinia* phage fHe-Yen3-01 that we recently isolated in Finland (Jun et al., 2018), followed by *Pectobacterium* phage MA13 (75,5%) and *Cronobacter sakazakii* phage vB_CskP_GAP227 (73,5%) (Table 1). Whole-genome phylogenetic tree (Figure 2) places f80-18 in well-defined clade, which was defined as *Gap227virus* (Kajsík et al., 2019) or broader as Ahp1-like subgroup (Wang et al., 2016). This significant phylogenetic association is supported by a tree inferred using single marker, RNA polymerase (RNAP) (Figure 3). Genome alignment of selected phages from this clade (Figure 4) revealed that most of the similarities (local sequence identity ≥ 60%) come from the predicted genes coding for DNA helicase (*g20*), DNA polymerase (*g28*), phosphoesterase (*g34*), RNA polymerase (*g37*), phage collar (*g42*), major capsid protein (*g44*), lytic glycosylase (*g49*) and DNA packaging protein (*g53*). The major genomic diversity regions are located to the early gene and the tail fiber protein encoding gene (*g50*) that score the lowest local identity results (<60%). Notably, the genome of the nearly identical phage fHe-Yen3-01 differs from f80-18 mainly by the absence of the *g03* gene. On the other hand, the fHe-Yen3-01 possesses the gene *g29* that is not related to any ϕ80-18 genes. The only other major difference between the two phages resides in the N-terminal parts of their respective tail fiber proteins that are only 56% identical, explaining the distinct differences in the host ranges between these phages (Jun et al., 2018).

**TABLE 1:** Overview of 15 phages most closely related to phage ϕ80-18.

**Description**	**Genome size (Kb)**	**Query coverage**	***E*-Value**	**Identity-%**	**Accession no.**
**Yersinia phage f80-18 complete genome**	42.41	100,00%	0.0	100.00	NC_019911.2
Yersinia phage fHe-Yen3-01, complete genome	42.77	97,00%	0.0	98.31	KY318515.1
Pectobacterium phage MA13, partial genome	42.46	47,00%	0.0	75.54	MN509793.1
Cronobacter sakazakii phage vB_CskP_GAP227, complete genome	41.8	42,00%	0.0	73.48	KC107834.1
Cronobacter phage Dev-CD-23823 complete sequence	41.62	44,00%	0.0	71.70	LN878149.1
Pectobacterium phage PP2, complete genome	41.84	41,00%	0.0	72.05	KX756572.1
Yersinia phage vB_YenP_ISAO8, complete genome	41.45	33,00%	0.0	71.80	KT184661.1
Pectobacterium phage Arno160, complete genome	41.38	41,00%	0.0	71.11	MK053931.1
Yersinia phage phiR8-01 complete genome	42.09	32,00%	0.0	71.97	HE956707.2
Aeromonas phage 25AhydR2PP, complete genome	42.70	31,00%	0.0	70.46	MH179473.2
Aeromonas phage ZPAH7B, complete genome	30.79	28,00%	0.0	69.93	MK330684.1
Aeromonas phage ZPAH7, complete genome	30.79	28,00%	0.0	69.93	MH992513.1
Aeromonas phage phiAS7, complete genome	41.57	33,00%	0.0	70.00	JN651747.1
Salmonella phage vB_SpuP_Spp16, complete genome	41.83	32,00%	0.0	69.17	MG878892.2
Aeromonas phage LAh5, complete genome	41.99	19,00%	6,00E-170	68.19	MK838111.1

### Characterization of Bacteriophage ϕ80-18 Growth and Stability

**FIGURE 5:**
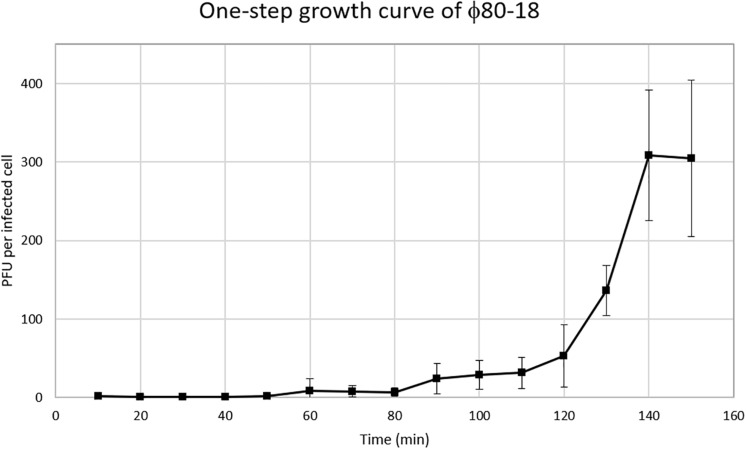
One-step growth curve of bacteriophage ϕ80-18.

To characterize the biological properties of ϕ80-18 its one-step growth curve was determined for the host strain *Y. enterocolitica* 8081-c (Figure 5). The phage seems to grow rather slowly in this host showing an apparent 50 min latent period, and low burst size of 8-10 PFU per infected bacterium. Comparing bacteriophage ϕ80-18 propagation at different temperatures similar effectivity was achieved at temperatures 4°C (4,0 × 10^8^ PFU/ml) and 28°C (8,7 × 10^7^ PFU/ml) and much lower efficiency at 37°C (7,3 × 10^4^ PFU/ml).

**FIGURE 6:**
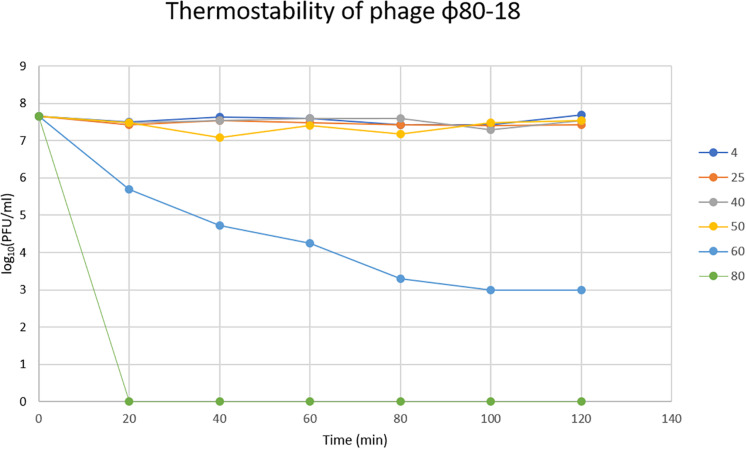
Thermostability of bacteriophage ϕ80-18 in temperature range 4–80°C.

**FIGURE 7:**
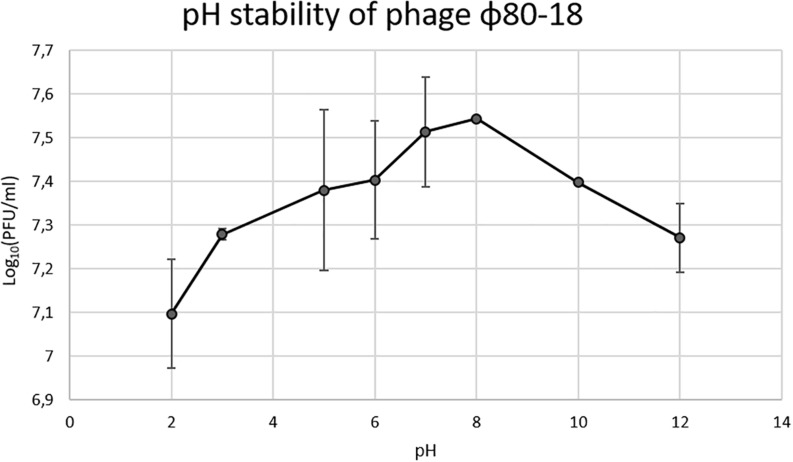
pH stability of bacteriophage ϕ80-18.

In the thermostability test the phage was stable for 2 h between +4 and 50°C, and was slowly inactivated at 60°C the titer dropping one log every 20 min, however, at 80°C it was completely inactivated already after 20 min incubation (Figure 6). The phage tolerated well pH values between 2 and 12 and had apparently optimal pH of 7-8 (Figure 7).

### Morphology of Bacteriophage ϕ80-18

**FIGURE 8:**
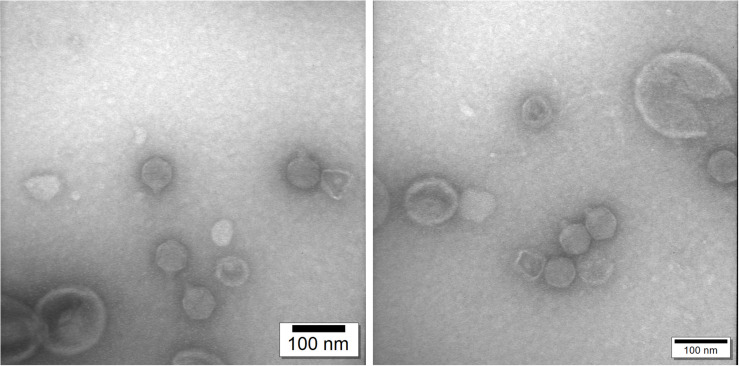
Transmission electron microscopy confirms that phage ϕ80-18 belongs to *Podoviridae* family of viruses.

Genome sequence and phylogenetic analysis showed that ϕ80-18 belongs to *Podoviridae* family of bacteriophages. Transmission Electron Microscopy confirmed that this phage has icosahedral capsid and short non-contractile tail with tail fibers. The dimensions of the phage are 59.0 ± 2.28 nm (*n* = 14) for capsid vertex to vertex, 59.0 ± 2.9 nm (*n* = 14) for capsid face to face and 18.3 ± 1.44 nm long tail (Figure 8).

### Host Range

The host range of phage ϕ80-18 was tested using 115 *Yersinia* strains representing *Y. aleksiciae, Y. bercovieri*, *Y. enterocolitica*, *Y. frederiksenii*, *Y. intermedia*, *Y. kristensenii*, *Y. mollaretii*, *Y. nurmii*, *Y. pekkanenii*, *Y. ruckeri*, and *Y. pseudotuberculosis* (Supplementary Table S1). Bacteriophage ϕ80-18 was able to infect 16 strains. The host range analysis showed that ϕ80-18 can infect in addition to *Y. enterocolitica* serotype O:8 strains also strains of serotypes O:4, O:4,32, O:20 and O:21, the latter ones representing similar to serotype O:8 the American pathogenic *Y. enterocolitica* serotypes. In addition, also strains of the non-pathogenic serotype O:7,8 and two of the bioserotype 1A/O:5 strains were infected by ϕ80-18 (Supplementary Table S1). The LPS O-antigen composed of pentasaccharide repeat units was shown to function as a receptor for phage ϕ80-18 (Zhang et al., 1997), and a very similar structure is present in serotype O:7,8 O-antigen, however, the O-antigen repeat unit structure of O:4,32 shares only the reducing-end sugar, *N*-acetylgalactosamine, with the O:8 structure (Skurnik and Zhang, 1996). The structures of serotype O:20 and O:21 O-repeat units are not known, however, it is possible that they also contain an O-unit with a reducing-end *N*-acetylgalactosamine. If so, the phage receptor structure could be composed of the junction between the LPS core and the reducing-end *N*-acetylgalactosamine of the O-antigen.

## Discussion

Foodborne illnesses are still common despite of the use of many antibacterial methods during food production such as pasteurization, high pressure processing (HPP), irradiation or chemical disinfectants. *Y. enterocolitica* is a food-borne zoonotic pathogen which is able to grow at 4°C, making it dangerous when contaminated product is stored at low temperatures. The most common source of this pathogen is raw pork (Leon-Velarde et al., 2019).

We show here that bacteriophage ϕ80-18 is stable and active in a wide range of pH (from 2 to 12) (Figure 7) and temperature (from 4 to 50°C) (Figure 6). These properties of ϕ80-18 make it a potential candidate for further research on the elimination of *Y. enterocolitica* serotype O:8 and possible other American serotypes, for example, during the processing of food products. And these properties suggest that the phage would be easy to maintain and store for longer periods. Furthermore, the tolerance to pH 2 further indicates that the phage particles might survive the exposure to gastric juices after oral administration of the phage. The tail fiber of phage ϕ80-18 is also a good candidate to be used for specific detection of the American pathogenic *Y. enterocolitica* serotype bacteria.

Phylogenetic trees constructed using the whole genome sequence (Figure 2) or RNAP protein sequence (Figure 3) confirmed that ϕ80-18 belongs to *Autographivirinae* subfamily in *Podoviridae* and shows the highest similarity to *Yersinia* phage fHe-Yen3-01 (Table 1), followed by *Pectobacterium* phage MA13, *Cronobacter sakazakii* phage vB_CskP_GAP227, *Cronobacter* phage Dev-CD-23823, *Pectobacterium* phage PP2 and *Pectobacterium* phage Arno160. Including other closely related phages such as *Yersinia* phage PhiR8-01, *Aeromonas* phage ZPAH7, *Salmonella* phage vB_SpuP_Spp16 and *Aeromonas* phage Ahp1, it forms a cluster that is stable in both phylogenetic analyses (Figure 2).

We recently demonstrated that *Yersinia* bacteriophage fHe-Yen3-01 can be used to treat of kitchen utensils (wooden and plastic cutting boards, knives) and artificial hands contaminated by *Y. enterocolitica* (Jun et al., 2018). After treatment with the phages, CFU counts remained constant for the first 2 h of the experiment. However, after 2 h, there were no detectable bacteria. The results of this experiments proves the potential of using *Yersinia* phages in the food industry. Phage fHe-Yen3-01 is closely related to phage ϕ80-18 (92% of nucleotide sequence identity) indicating that ϕ80-18 is also a good candidate for this type of research.

Additionally, the research on the PY100 phage was interesting because of its lytic properties and activity in controlling *Yersinia* in meat. PY100 significantly reduced the number of bacteria at 4°C in pork (the best results were obtained at a MOI 10^4^, when the number of bacteria decreased by up to 5 log_10_ units) (Orquera et al., 2012). This is also an encouraging argument for the possibility of using *Yersinia* phages in the food industry.

The food poisoning is still one of the major causes of hospitalization or even patients death around the world (Moye et al., 2018). Pasteurization and HPP, are methods used for inactivating microbes in liquids, dairy products and pre-cooked meals. However, these methods cannot be used with fresh meats due to their influence on the color as well as the nutritional content (Wolbang et al., 2008; Bajovic et al., 2012; Moye et al., 2018).

Irradiation is effective in reducing pathogenic bacteria in food, but it can also affect the food’s organoleptic properties. Chemical sanitizers, such as chloride, reduce bacteria from fruits and vegetables surface, but also these chemicals affect the environment (Beuchat and Ryu, 1997; Sohaib et al., 2016; Moye et al., 2018). More consumers now do not tolerate chemical additives in foods for example, because of allergies. However, all the microbes (pathogenic bacteria, normal flora or probiotic bacteria) are killed by all these methods.

A completely another approach is to use lytic bacteriophages for specific foodborne bacteria in foods thereby circumventing any adverse influence on normal, most of the time beneficial microflora. Currently, phage biocontrol is the most environmentally friendly method which can be used to eradicate pathogens from food products (Moye et al., 2018). At present, several phages have been approved by the FDA for use in the food industry.

Phages are used in the food industry to combat pathogens such as *E. coli* 0157:H7, *Listeria monocytogenes*, *Salmonella* spp., *Shigella* spp. (Moye et al., 2018). These are the first steps toward using lytic bacteriophages as safe and natural antibacterial agents. Bacteriophage biocontrol can be used both pre-harvest (e.g., live animals) and post-harvest (e.g., applied to food surface, packing materials) to remove pathogens.

## Data Availability Statement

The datasets presented in this study can be found in online repositories. The names of the repository/repositories and accession number(s) can be found here: https://www.ncbi.nlm.nih.gov/genbank/, HE956710.

## Author Contributions

BS-O performed the stability experiments and took TEM photography of the phage. KF took part in conducting experiments. LH and AN performed the initial annotation. MS performed the final annotation of the genome and analyzed the data. MP purified and prepared the phage particles for proteomic analysis. MQ determined the physical ends of the genome. JJ and MS carried out the host range analyses. LM prepared the phage genome for Illumina sequencing and carried out the initial genome *de novo* assembly of the genome. EB and MS supervised the studies. KF and MW performed the phylogenetic and genome analysis. All authors read and approved the final manuscript.

## Conflict of Interest

The authors declare that the research was conducted in the absence of any commercial or financial relationships that could be construed as a potential conflict of interest.

## Footnotes

1 http://www.softgenetics.com
