# Supplementary material for: The Podovirus ϕ80-18 Targets the Pathogenic American Biotype 1B Strains of Yersinia enterocolitica
Source: Front Microbiol. 2020 Jun 19;11:1356. doi: 10.3389/fmicb.2020.01356 (PMC7316996; doi:10.3389/fmicb.2020.01356)
Supplement: Supplementary file 1 [file Data_Sheet_1.pdf]

SUPPLEMENTARY TABLES

The podovirus ϕ80-18 targets the pathogenic American biotype 1B strains of *Yersinia enterocolitica*

Karolina, Filik[1#], Bożena Szermer-Olearnik[1#], Maciej Wernecki[2], Lotta J. Happonen[3,7], Maria I. Pajunen[4], Ayesha Nawaz[4], Suleman Mohammed Qasim[4], Jin Woo Jun[5], Laura Mattinen[4], Mikael Skurnik[4,6#] and Ewa Brzozowska[1]

[1] *Hirszfeld Institute of Immunology and Experimental Therapy, Polish Academy of Sciences, Wroclaw, Poland*

[2] *Department of Microbiology, Institute of Genetics and Microbiology, University of Wroclaw, Wroclaw, Poland*

[3] *Institute of Biotechnology and Department of Biosciences, University of Helsinki, Helsinki, Finland.*

[4] *Department of Bacteriology and Immunology, Human Microbiome Research Program, Faculty of Medicine, University of Helsinki, Helsinki, Finland*

[5] *Department of Aquaculture, Korea National College of Agriculture and Fisheries, Jeonju 54874, Republic of Korea*

[6] *Division of Clinical Microbiology, Helsinki University Hospital, HUSLAB, Helsinki, Finland*

[7] *Lund University, Department of Clinical Sciences Lund, Infection Medicine, Lund, Sweden.*

[#] Coressponding authors:

E-mails:
mikael.skurnik@helsinki.fi;
karolina.filik@hirszfeld.pl;
bożena.szermer-olearnik@hirszfeld.pl

Table S1 List of *Yersinia* strains used in phage ϕ80-18 host range experiments. All the strains are from the Skurnik lab strain collection. Efficiency of plating (EOP) with strain 8081 set as 1.0.

Bacterial Strain	Origin / Characteristics	Serotype[a]	Stor ID	Phage sensitivity (EOP)	Reference
Yersinia aleksiciae					
404/81	Human, Finland	O:16	284	-	[1]
317/82	Human, Finland	O:16	390	-	
Yersinia bercovieri					
3016/84	Human, Finland	O:58,16	781	-	[2]
127/84	Human, Finland	NT	789	-	[2]
3984/84	Human, Finland	O:58,16	790	-	[2]
Yersinia enterocolitica					
10/84	Human, Finland	NT	760	-	[3]
1539	Human, Finland	NT	286	-	[3]
8533/84	Human, Finland	NT	841	-	[3]
659/83	Human, Finland	K1, NT	741	-	[3]
4367/83	Human, Finland	K1, NT	533	-	[3]
gk132	Chinchilla	O:1	908	-	[4]
JDE029	Human	O:1	1006	-	[5]
gk2943	Goat, Norway	O:2	942	-	[3]
gk1142	Hare, Norway	O:2	943	-	[6]
20373/79	Human, Finland	O:3	2	-	[3]
5854	Human, Finland	O:3	3	-	[3]
9568/79	Human, Finland	O:3	4	-	[3]
6471/76 (YeO3)	Human, Finland	O:3	1254	-	[7]
6471/76-c (YeO3-c)	Human, Finland	O:3	1255	-	[7]
YeO3-c-OC	LPS mutant, derivative of YeO3-c	O:3	2477		[8]
YeO3-c-OCR	LPS mutant, derivative of YeO3-c	O:3 (rough)	2684		[8]
JDE766	Human, North America	O:1,2,3	1002	-	[5]
gc3973-76	Stool, USA	O:4	1029	+ (0.23±0.05)	[9]
JDE701	Human, North America	O:4,32	952	+++ (3.73±0.69)	[10]
4209 CR+	Unknown, Canada	O:5	968	+	[11]
14693/84	Human, Finland	O:5	876	-	[3]
298/85a2	Human, Finland	O:5	908	+	[3]
20109/83	Human, Finland	O:5	730	-	[3]
14779/83	Human, Finland	O:5	664	-	[4]
17223/83	Human, Finland	O:5	693	-	[3]
18710/83	Human, Finland	O:5	709	-	[3]
gk7500	Coypu, Unknown	O:5,27	944	-	[6]
JDE657	Human, North America	O:5,27	1004	-	[5]
JDE654	Human, North America	O:5,27	1007	-	[5]
gc815-73	Unknown, USA	O:5,27	1026	-	[9]
590/80	Human, Finland	O:6	96	-	[7]

Bacterial Strain	Origin / Characteristics	Serotype[a]	Stor ID	Phage sensitivity (EOP)	Reference
266/84	Human, Finland	O:6	838	-	[3]
189/80	Human, Finland	O:6,30	92	-	[12]
6737/80	Human, Finland	O:6,30	200	-	[12]
3604/80	Human, Finland	O:6,30	201	-	[12]
438/80	Human, Finland	O:6,31	217	-	[7]
1309/80	Human, Finland	O:6,31	16	-	[7]
605	Human, Finland	O:7,8	98	+	[7]
22848/79	Human, Finland	O:7,8	28	+	[7]
17869/83	Human, Finland	O:7,8	698	+++ (3.37±0.67)	[3]
p310	Unknown	O:8	252	+	[3]
CDCA2635	Milk, USA	O:8	277	+	[13]
TAMU-75	Human, USA	O:8	278	+	[13]
WA	Human, USA	O:8	322		[13]
JDE661	Human, North America	O:8	999	+	[5]
8081 (YeO8)	Human septicemia, USA	O:8	1258	++ (1.00)	[14]
8081-c	pYV-cured derivative of 8081	O:8	1259	+++ (3.03±0.12)	[14]
8081-R2	Rough derivative of 8081	rough O:8	1978		[15]
8081-c-R2	Rough derivative of 8081-c	rough O:8	1978		[15]
YeO8-c:: ΔwbcEGB	LPS mutant of 8081-c	Semi-rough O:;8	2427		[16]
YeO8::Δwzz GB	LPS mutant of 8081		2429		[16]
YeO8:: Δwbc EGB	LPS mutant of 8081	Semi-rough O:;8	2446		[16]
277/74	Human, Finland	O:9	1	-	[3]
4945/74	Human, Finland	O:9	21	-	[3]
767/73	Human, Finland	O:9	54	-	[3]
467/73	Human, Finland	O:9	55	-	[17]
3672/74	Human, Finland	O:9	97	-	[3]
13752/73	Human, Finland	O:9	137	-	[3]
Ruokola/71-c	Human, Finland	O:9	1257	-	[7]
3102/80	Human, Finland	O:10	102	-	[12]
3788/80	Human, Finland	O:10	210	-	[12]
2640/84	Human, Finland	O:10	777	-	[3]
10927/84	Human, Finland	O:10	851	-	[3]
gc1209-79	Blood, USA	O:13	1025	-	[9]
ST5081	Unknown, USA	O:13a,13b	1047	-	[18]
421/84	Human, Finland	O:13,7	862	-	[3]
2446/84	Human, Finland	O:13,7	775	-	[3]
gc9312-78	Stool, USA	O:13,18	1023	-	[9]
15712/83	Human, Finland	O:14	679	-	[1]
gc874-77	Stool, USA	O:20	1021	+	[9]
gc1223-75	Stool, USA	O:20	1028	+++ (6.82±0.85)	[9]
E736	North America	O:21	953	-	[10]
WI-81-50	North America	O:21	956	+ (0.32±0.10)	[19]
80-EA-63	North America	O:21	957	-	[19]

Bacterial Strain	Origin / Characteristics	Serotype[a]	Stor ID	Phage sensitivity (EOP)	Reference
431/84	Human, Finland	O:25	878	-	[1]
63/84	Human, Finland	O:26,44	768	-	[20]
18425/83	Human, Finland	O:25,26,44	704	-	[20]
5186/84	Human, Finland	O:28,50	813	-	[20]
gc2139-72	Unknown, USA	O:34	1027	-	[9]
248/84	Human, Finland	O:35,52	824	-	[20]
7104/83	Human, Finland	O:35,36	568	-	[20]
264/85	Human, Finland	O:41,43	904	-	[1]
626/83	Human, Finland	O:41(27),42	728	-	[1]
9613/83	Human, Finland	O:41(27), K1	590	-	[3]
19942/83	Human, Finland	O:41(27),42, K1	729	-	[20]
1346/84	Human, Finland	O:41(27),43	761	-	[20]
647/83	Human, Finland	O:41(27),43	740	-	[20]
3229	Human, Finland	O:50	209	-	[4]
Yersinia frederiksenii					
38/83	Human, Finland	O:48	502	-	[1]
3400/83	Human, Finland	O:16	532	-	[1]
3317/84	Human, Finland	O:35	785	-	[1]
498/85	Human, Finland	NT	910	-	[21]
28/85	Human, Finland	K1, NT	915	-	[21]
IP23047	Institut Pasteur, France	O:3	2287	-	[22]
Yersinia intermedia					
9/85	Human, Finland	O:16,21	914	-	[1]
821/84	Human, Finland	O52,54	757	-	[4]
Yersinia kristensenii					
4336/83	Human, Finland	UT	535	-	[3]
19602/83	Human, Finland	NT	720	-	[3]
119/84	Human, Finland	O:12,25	783	-	[4]
IP22828	Institut Pasteur, France	O:3	2288	-	[22]
Yersinia mollaretii					
92/84	Human, Finland	O:59(20,36,7)	778	-	[2]
IP22404	Institut Pasteur, France	O:3	2289	-	[22]
Yersinia nurmii					
DSM 22296	Meat, Finland	UT	5607	-	[23]
Yersinia pekkanenii					
Å125KOH2	Lettuce, Finland	UT	3059	-	[24]
Yersinia pseudotuberculosis					
2812/79	Human, Finland	O:1b	192	-	[7]
677/82	Human, Finland	O:1b	407	-	[3]
324/80	Human, Finland	O:3	214	-	[4]
1261/79	Human, Finland	O:3	245	-	[7]
Yersinia ruckeri					
OMBL3	Fish isolate, Finland	UT	884	-	[4]
OMBL4	Fish isolate, Finland	UT	885	-	[21]

[a] NT, non-typeable; UT, untyped.

REFERENCES

1. Skurnik, M.; Toivonen, S. Identification of distinct lipopolysaccharide patterns among Yersinia enterocolitica and Y. enterocolitica-like bacteria. *Biochemistry (Mosc)* **2011**, *76*, 823-831.

2. Wauters, G.; Janssens, M.; Steigerwalt, A.D.; Brenner, D.J. Yersinia mollaretii sp. nov. and Yersinia bercovieri sp. nov., formerly called Yersinia enterocolitica biogroups 3A and 3B. *Int J Syst Bacteriol* **1988**, *38*, 424-429.

3. Leon-Velarde, C.G.; Happonen, L.; Pajunen, M.; Leskinen, K.; Kropinski, A.M.; Mattinen, L.; Rajtor, M.; Zur, J.; Smith, D.; Chen, S.*, et al. Yersinia enterocolitica*-specific infection by bacteriophages TG1 and ϕR1-RT is dependent on temperature-regulated expression of the phage host receptor OmpF. *Appl Environ Microbiol* **2016**, *82*, 5340-5353.

4. Skurnik, M.; Toivanen, P. Intervening sequences (IVSs) in the 23S ribosomal RNA genes of pathogenic *Yersinia enterocolitica* strains. The IVSs in *Y. enterocolitica* and *Salmonella typhimurium* have common origin. *Mol. Microbiol.* **1991**, *5*, 585-593.

5. Schiemann, D.A.; Devenish, J.A. Relationship of HeLa cell infectivity to biochemical, serological, and virulence characteristics of *Yersinia enterocolitica*. *Infect. Immun.* **1982**, *35*, 497-506.

6. Kapperud, G.; Skarpeid, H.-J.; Solberg, R.; Bergan, T. Outer membrane proteins and plasmids in different Yersinia enterocolitica serogroups isolated from man and animals. *Acta path microbiol immunol scand Sect B* **1985**, *93*, 27-35.

7. Skurnik, M. Lack of correlation between the presence of plasmids and fimbriae in *Yersinia enterocolitica* and *Yersinia pseudotuberculosis*. *J. Appl. Bact.* **1984**, *56*, 355-363.

8. Biedzka-Sarek, M.; Venho, R.; Skurnik, M. Role of YadA, Ail, and lipopolysaccharide in serum resistance of *Yersinia enterocolitica* serotype O:3. *Infect. Immun.* **2005**, *73*, 2232-2244.

9. Kay, B.A.; Wachsmuth, K.; Gemski, P.; Feeley, J.C.; Quan, T.J.; Brenner, D.J. Virulence and phenotypic characterization of *Yersinia enterocolitica* isolated from humans in the united states. *J. Clin. Microbiol.* **1983**, *17*, 128-138.

10. Perry, R.D.; Brubaker, R.R. Vwa^+ phenotype of *Yersinia enterocolitica*. *Infect. Immun.* **1983**, *40*, 166-171.

11. Prpic, J.K.; Robins-Browne, R.M.; Davey, R.B. Differentiation between virulent and avirulent *Yersinia enterocolitica* isolates by using congo red agar. *J. Clin. Microbiol.* **1983**, *18*, 486-490.

12. Skurnik, M.; Nurmi, T.; Granfors, K.; Koskela, M.; Tiilikainen, A.S. Plasmid associated antibody production against *Yersinia enterocolitica* in man. *Scand. J. Inf. Dis.* **1983**, *15*, 173-177.

13. Gemski, P.; Lazere, J.R.; Casey, T. Plasmid associated with pathogenicity and calcium dependency of *Yersinia enterocolitica*. *Infect Immun* **1980**, *27*, 682-685.

14. Portnoy, D.A.; Falkow, S. Virulence-associated plasmids from *Yersinia enterocolitica* and *Yersinia pestis*. *J. Bacteriol.* **1981**, *148*, 877-883.

15. Zhang, L.; Radziejewska-Lebrecht, J.; Krajewska-Pietrasik, D.; Toivanen, P.; Skurnik, M. Molecular and chemical characterization of the lipopolysaccharide O-antigen and its role in the virulence of *Yersinia enterocolitica* serotype O:8. *Mol. Microbiol.* **1997**, *23*, 63-76.

16. Bengoechea, J.A.; Najdenski, H.; Skurnik, M. Lipopolysaccharide O antigen status of *Yersinia enterocolitica* O:8 is essential for virulence and absence of O antigen affects the expression of other *Yersinia* virulence factors. *Mol. Microbiol.* **2004**, *52*, 451-469.

17. Kiljunen, S.; Hakala, K.; Pinta, E.; Huttunen, S.; Pluta, P.; Gador, A.; Lönnberg, H.; Skurnik, M. Yersiniophage ϕR1-37 is a tailed bacteriophage having a 270 kb DNA genome with thymidine replaced by deoxyuridine. *Microbiology* **2005**, *151*, 4093-4102.

18. Toma, S.; Wauters, G.; McClure, H.M.; Morris, G.K.; Weissfeld, A.S. O:13a,13b, a new pathogenic serotype of Yersinia enterocolitica. *J Clin Microbiol* **1984**, *20*, 843-845.

19. Schiemann, D.A. Antigenic identity of *Yersinia enterocolitica* serotypes O:Tacoma and O21. *J Clin Microbiol* **1984**, *20*, 831-832.

20. Skurnik, M. Studies on the virulence plasmids of *Yersinia* species. PhD, University of Oulu, Oulu, 1985.

21. Reuter, S.; Connor, T.R.; Barquist, L.; Walker, D.; Feltwell, T.; Harris, S.R.; Fookes, M.; Hall, M.E.; Petty, N.K.; Fuchs, T.M.*, et al.* Parallel independent evolution of pathogenicity within the genus Yersinia. *Proc Natl Acad Sci U S A* **2014**, *111*, 6768-6773.

22. Pajunen, M.; Kiljunen, S.; Skurnik, M. Bacteriophage ϕYeO3-12, specific for *Yersinia enterocolitica* serotype O:3, is related to coliphages T3 and T7. *J. Bacteriol.* **2000**, *182*, 5114-5120.

23. Murros-Kontiainen, A.E.; Fredriksson-Ahomaa, M.; Korkeala, H.; Johansson, P.; Rahkila, R.; Björkroth, J. *Yersinia nurmii* sp. nov. *Int. J. Sys. Evol. Microbiol.* **2011**.

24. Murros-Kontiainen, A.E.; Johansson, P.; Niskanen, T.; Fredriksson-Ahomaa, M.; Korkeala, H.; Björkroth, J. *Yersinia pekkanenii* sp. nov. *Int. J. Sys. Evol. Microbiol.* **2011**.

Table S2. Predicted genes and gene products of bacteriophage ϕ80-18 (acc. no. HE956710).

Gene		Gene product			BLAST and Hhpred similarity searches				Predicted function
**Name**	**Location**	**Size (aa)[2]**	**Molecular mass (kDa)[2]**	**pI[3]**	**PSI-BLAST[4] (id-%)**	**Organism**	**Sequence ID**	**Hhpred domain search[5] Probability % / e-value**	
g01	933-1283	116	13.4	10.2	MA13_gp04 (72%)	Pectobacterium phage MA13	QGF20948.1	No hits	hypothetical protein
g02	1857-2414	185	20.4	8.5	PP2_002 (66 %)	Pectobacterium phage PP2	AOT25368.1	No hits	hypothetical protein
g03	2411-2602	63	7.1	4.3	No hits			cd13329; Rho guanine nucleotide exchange factor Pleckstrin homology domain 67.57 / 5.1	hypothetical protein
g04	2648-2941	97	10.8	5.6	Gp1.05	Escherichia phage IMM-002	ATI16975.1	No hits	hypothetical protein
g05	2931-3164	77	9.2	7.6	73 aa protein (36%)	Pantoea phage vB_PagP-SK1	QFR42365.1	No hits	hypothetical protein
g06	3164-3397	77	8.7	9.6	BN110_019 (55%)	Yersinia phage phiR8-01	CC188389.2	No hits	hypothetical protein
g07	3464-3691	75	8.6	9.2	Arno160_gp08	Pectobacterium phage Arno160	AZF88070.1	No hits	hypothetical protein
g08	3688-4497	269	29.8	9.0	Only hit fHeYen301_7 (73%)	Yersinia phage fHe-Yen3-01	APU00340.1	cd03819; GT4 family glycosyltransferase 72.81 / 5.1	hypothetical protein
g09	4494-4886	130	14.5	5.4	MA13_gp08 (44%)	Pectobacterium phage MA13	QGF20952.1	cd16380; Bacillus subtilis YitT domain 90.48 / 0.95 cd00953; archeal 2-keto-3-deoxygluconate aldolase 74.99 / 10	**PPAP**
g10	4867-5097	76	8.5	9.4	BN110_029 (53%)	Yersinia phage phiR8-01	CC188400.2	No hits	hypothetical protein
g11	5081-5434	117	12.8	9.9	EJ02DRAFT_439484 (33%)	Clathrospora elynae	KAF1935050.1	No hits	hypothetical protein
g12	5434-5916	160	17.3	5.4	GAP227_11 (38%)	Cronobacter phage vB_CskP_GAP 227	YP_007348330.1	No hits	**PPAP**
g13	5916-6149	77	8.7	5.6	HRP29_gp12 (30%)	Shigella phage HRP29	QBP32912.1	No hits	hypothetical protein
g14	6146-6370	74	8.4	6.0	HRP29_gp12 (33%)	Shigella phage HRP29	QBP32912.1	No hits	hypothetical protein
g15	6367-6501	44	4.8	8.7	Only hit fHeYen301_14 (73%)	Yersinia phage fHe-Yen3-01	APU00347.1	No hits	hypothetical protein
g16	6515-6766	83	9.4	9.4	BN110_026 (53%)	Yersinia phage phiR8-01	CC188396.2	No hits	**PPAP**
g17	6768-6962	64	7.8	10.1	Only hit fHeYen301_16 (98%)	Yersinia phage fHe-Yen3-01	APU00349.1	No hits	hypothetical protein

g18	6978-7694	238	27.2	8.9	DNA primase (54%)	Pectobacterium phage Arno160	AZF88076.1	cd03364; DnaG primase nucleotidyltransferase/hydrolase domain 92.98 / 0.19	**DNA primase/helicase, PPAP**
g19	7691-7885	64	7.7	10.1	Only hit fHeYen301_18 (38%)	Yersinia phage fHe-Yen3-01	APU00351.1	cd09804; mRNA decapping enzyme 72.52 / 4.2	hypothetical enzyme
g20	7879-9123	414	46.2	5.9	DNA helicase (76%)	Cronobacter phage vB_CskP_GAP 227	YP_007348335. 1	cd01122; homohexameric 5'-3' helicase 99.85 / 2.3e-19	**DNA helicase; PPAP**
g21	9140-9268	42	4.5	4.7	Only hit fHeYen301_20 (95%)	Yersinia phage fHe-Yen3-01	APU00353.1	No hits	hypothetical protein
g22	9225-9476	83	9.2	5.3	MP2_gp20 (35%)	Morganella phage vB_MmoP_MP 2	YP_009291546. 1	cd08769; peptidase 34.3 / 10	hypothetical peptidase
g23	9469-9651	60	6.6	4.6	hypothetical protein (25%)	Salmonella phage vB_SpuP_Spp1 6	AVI05053.1	cd15485; leucine zipper domain 72.18 / 0.93	hypothetical protein
g24	9644-9841	65	7.7	5.6	E5A41_05345 (44%)	Salmonella enterica	KAA6658853.1	No hits	hypothetical protein
g25	9841-10800	319	36.4	6.8	DNA ligase (52%)	Pectobacterium phage MA13	QGF20957.1	cd07902; Adenylation DNA ligase 99.88 / 2.4e-21	**DNA ligase; PPAP**
g26	10556-10939	127	14.6	6.4	No hits			cd18525; BACK domain 46.0 / 11	hypothetical protein
g27	10917-11681	254	29	4.6	nucleotidyl transferase (20%)	Erwinia phage vB_EamP-S2	AUV57215.1	cd05398; nucleotidyltransferase domain 99.8 / 2.8e-19	**Putative nucleotidyltransferase; PPAP**
g28	11694-14147	817	92.6	5.6	DNA polymerase (82%)	Pectobacterium phage MA13	QGF20958.1	cd06139; 3'-5' exonuclease 99.07 / 1e-9 cd08637; DNA polymerase 100.0 / 1.8e-49	**DNA polymerase; PPAP**
g29	14147-14338	63	6.7	4.6	Sodium channel protein type 5 subunit alpha (28%)	Tetrabaena socialis	PNH09896.1	cd08962; Glu-tRNA amidotransferase 40.23 / 22	hypothetical amidotransferase
g30	14355-15179	274	29.7	4.8	MA13_Gp15 (71%)	Pectobacterium phage MA13	QGF20959.1	No hits	**PPAP**
g31	15179-16177	332	37.9	6.7	DNA exonuclease (73 %)	Pectobacterium phage PP2	AOT25387.1	cd00008; 5'-3' exonuclease, T5-5' nuclease 99.86 / 7e-22	**5'-exonuclease; PPAP**
g32	16174-16599	141	15.6	4.6	MA13_Gp17 (54%)	Pectobacterium phage MA13	QGF20961.1	No hits	**PPAP**
g33	16475-16921	148	16.5	9.9	putative DNA endonuclease (67%)	Aeromonas phage 25AhydR2PP	AWH15420.1	cd12870; MqsA antitoxin of MqsR 58.9 / 4.1	**Phage endonuclease; weak similarity to antitoxin**
g34	16921-17886	321	35.6	7.8	BN110_037 (82%)	Yersinia phage phiR8-01	CC188408.2	cd07424; metallophosphatase domain of type I serine/threonine and tyrosine phosphatases 99.46 / 3.5e-14	**Metallophosphatase; PPAP**
g35	17887-18063	58	6.8	6.1	putative ATP-dependent RNA helicase (31%)	Trypanosoma rangeli	XP_029241962. 1	cd16127; ubiquitin-like domain 37.02 / 10	hypothetical protein

g36	18044-18691	215	24.5	5.4	Tonnikala_41 (35%)	Escherichia phage tonnikala	QHR71312.1	cd02022; DPCK 98.49 / 4.2e-8 cd00227; CPT 98.31 / 2.7e-6 cd01672; TMPK 97.72 / 1.1e-5 cd02023; UMPK 97.47 / 3.4e-5	**Kinase phoshorylating CoA, chloramphenicol, TMP or uridine; PPAP**
g37	18702-21155	817	92.7	7.8	Phage RNA polymerase (65%)	Cronobacter phage Dev-CD-23823	YP_009223403.1	cd08642; DNA polymerase 92.66 / 0.5	**DNA-directed RNA polymerase**
g38	21249-21410	53	5.8	8.3	PP2_029 (73 %)	Pectobacterium phage PP2	AOT253395.1	cd14765; hemoglobin 67.12 / 5.5	hypothetical protein
g39	21407-21832	141	16.0	8.8	Hypothetical protein (65%)	Cronobacter phage Dev-CD-23823	YP_009223405.1	cd04301; N-acyltransferase superfamily 98.66 / 2.1e-7 cd02169; citrate lyase ligase acetylates CoA 98.3 / 3.1e-6	**N-acyltransferase or acetyltransferase**
g40	21832-22227	131	14.1	9.3	Hypothetical protein CF7_11 (51%)	Aeromonas phage CF7	ASZ71957.1	No hits	**PPAP**
g41	22227-22406	59	6.4	4.6	Anti-sigma regulatory factor (33%)	Fortiea contorta	WP_017652703.1	No hits	hypothetical protein
g42	22415-23920	501	56.2	5.0	Putative head portal protein (71%)	Aeromonas phage phiAS7	YP_007007810.1	No hits	**Phage collar; PPAP**
g43	23923-24729	268	28.0	4.6	Scaffolding protein (55%)	Pectobacterium phage MA13	QGF20968.1	No hits	**Scaffolding-like protein; PPAP**
g44	24804-25820	338	36.8	5.3	Major capsid protein (83%)	Pectobacterium phage MA13	QGF20969.1	No hits	**Major capsid protein; PPAP**
g45	25917-26510	197	22.1	6.4	Putative tail tubular protein A (62%)	Aeromonas phage phiAS7	YP_007007807.1	No hits	**Tail tubular protein A; PPAP**
g46	26514-29123	869	97.6	5.8	Putative tail tubular protein B (60%)	Aeromonas phage phiAS7	YP_007007806.1	No hits	**Phage tail fiber protein; PPAP**
g47	29125-29892	255	27.3	6.3	hypothetical protein (47%)	Aeromonas phage 25AhydR2PP	AWH15406.1	No hits	**PPAP**
g48	29903-32152	749	82.0	5.6	Hypothetical protein (53%)	Cronobacter phage Dev-CD-23823	YP_009223413.1	No hits	**PPAP**
g49	32156-35935	1259	137.5	5.7	Lytic glycolase (62%)	Cronobacter phage vB_CskP_GAP227	YP_007348360.1	cd13403; lytic murein transglycolase 98.63 / 9.2e-8	**Lytic transglycosylase; PPAP**
g50	36005-38014	669	70.8	5.0	Putative tail fiber protein (23%)	Aeromonas phage phiAS7	YP_007007802.1	No hits	**Phage tail fiber protein; PPAP**
g51	38029-38232	67	7.3	9.2	Holin (52 %)	Pectobacterium phage PP2	AOT25407.1	cd14263; diacylglycerol kinase 32.86 / 35	hypothetical kinase
g52	38213-38551	112	12.5	5.2	GAP227_42 (69%) Putative DNA maturase A or terminase small subunit	Cronobacter phage vB_CskP_GAP	YP_007348363.1	cd03460; Catechol 1,2 dioxygenase 53.15 / 20	**DNA packaging protein A, likely also PPAP**

						227			
g53	38551-40455	634	71.5	5.7	DNA maturase B (75 %) or terminase large subunit	Pectobacterium phage PP2	AOT25409.1	cd17921; DEXH-box helicase domain 98.57 / 1.9e-7 cd13962; Holliday junction resolvase RuvC 94.97 / 0.53	**DNA packaging protein B; PPAP**
g54	40483-40890	135	14.8	5.0	hypothetical protein (29%)	Salmonella phage vB_SpuP_Spp1 6	AVI05064.1	No hits	**PPAP**
g55	40926-41480	184	20.5	9.1	Phage lysin (65%)	Yersinia phage phiR8-01	CC188419.2	cd16900; endolysin R21 99.72 / 4.1e-16	**Lysozyme**
g56	41459-41839	126	13.8	5.6	Only hit fHeYen301_55 (95%)	Yersinia phage fHe-Yen3-01	APU00388.1	No hits	hypothetical protein
g57	41709-42002	97	10.8	6.6	Rz1-like lysis protein (47%)	Pectobacterium phage Arno160	AZF88111.1	No hits	Rz1-like lysis protein

[1] http://www.endmemo.com/bio/gc.php

[2] https://www.uniprot.org/

[3] https://web.expasy.org/compute_pi/

[4] The fHe-Yen3-01 hits were excluded from this column. In general, all ϕ80-18 gene products are >97% identical to corresponding ones in fHe-Yen3-01.

[5] NCBI conserved domains database v_3.17

Table S3. The predicted phage and host RNA polymerase promoters in the ϕ80-18 genome. The predicted -35 and -10 boxes of the host RNA polymerase promoters, and the BPROM scores of the boxes, are highlighted in yellow and green, respectively.

Promoter	Location (next gene)	Sequence			
**Phage RNA polymerase promoters**					
Phage P1	351-375 (*g01*)	CTGATTGTCTACCCATATAGTAACA			
Phage P2	21210-21234 (*g38*)	CTGATACTCTACCCATATAGCAACT			
Phage P3	24726-24750 (*g44*)	TTGATTGTCTACCCATATAGCAATA			
Phage P4	35959-35983 (*g50*)	TTGATTCTCTACCCATATAGTAACA			
	Consensus	-TGATt-TCTACCCATATAG-AAca			
**Host RNA polymerase promoters**		**-35 box**		**-10 box**	**Scores**
Host P5	741-769 (*g01*)	TTGACA	GCTTCAGAGTAACA	AGTTAGTAT	57 / 66
Host P6	1533-1561 (*g02*)	TGGCTG	GTTGAAACCCTCGG	CTATATAAA	33 / 10
Host P7	1785-1811 (*g02*)	TAGTCA	GTTGGTTAATGT	AAGTATCAT	57 / 16
Host P8	2873-2898 (*g05*)	TTTATC	TACAAGGATGCG	GATATATT	59 / 17
Host P9	3654-3687 (*g08*)	TGGAAT	CATACTGCCGTCGATTTGG	AGTTAAATT	61 / 18
Host P10	6883-6910 (*g18*)	TAGAAG	TCAAGCATAATGA	TGGTAAGGT	47 / 17
Host P11	11610-11638 (*g28*)	TTGAAA	GTACATGATGCACG	TCCTAATAT	53 / 60
Host P12	16130-16162 (*g32*)	TTGAAA	CAACAAGCTAAACTTAAC	TGGTTTATT	36 / 60
Host P13	26485-26513 (*g46*)	TTGGCA	ATCGTATTCCAGTG	AGGTAATAT	68 / 38
Host P14	38116-38147 (*g52*)	CTGCAA	GACTGGGTGTATATCTT	AACTATTA	55 / 20
Host P15	40853-40886 (*g55*)	CTGAAA	CAGAGCCAGCCGGTAAGAA	CGGTATCGT	48 / 25
